# Ulcerative Cutaneous Lesions Synchronously Present with the Diagnosis of Primary Lung Cancer

**DOI:** 10.1155/2013/136564

**Published:** 2013-03-07

**Authors:** Khaldoon Shaheen, Abdul Hamid Alraiyes, Motaz Baibars, Ama Paintsil, M. Chadi Alraies

**Affiliations:** ^1^Department of Hospital Medicine, Institute of Medicine, Cleveland Clinic Lerner College of Medicine of Case Western Reserve University, Cleveland Clinic, Cleveland, OH 44195, USA; ^2^Department of Pulmonary, Critical Care and Environmental Medicine, Tulane University, Health Sciences Center, New Orleans, LA 70112, USA; ^3^Hospital Medicine Section, Internal Medicine Department, Peninsula Regional Medical Center, Salisbury, MD 21801, USA; ^4^Internal Medicine Department, Case Western Reserve University, St. Vincent Charity Medical Center, Cleveland, OH 44115, USA

## Abstract

The percentage of patients with lung cancer that develop skin metastases is low. The diagnosis is usually made using clinical information and skin biopsy in patients with suspicious skin lesions and history of smoking or lung cancer. The prognosis for patients having lung cancer with skin metastasis is very poor. We describe findings in a 70-year-old man with lung cancer with skin metastases. Interestingly, multiple skin lesions were the first manifestation of the underlying lung cancer. The prognosis for patients having lung cancer with skin metastasis is thus very poor.

## 1. Introduction

The percentage of patients with lung cancer that develop skin metastases is low. The diagnosis is usually made using clinical information and skin biopsy in patients with suspicious skin lesions and history of smoking or lung cancer. The prognosis for patients having lung cancer with skin metastasis is very poor. We describe findings in a 70-year-old man with lung cancer with skin metastases. Interestingly, multiple skin lesions were the first manifestation of the underlying lung cancer. The prognosis for patients having lung cancer with skin metastasis is thus very poor.

## 2. The Case

**Figure 1:**
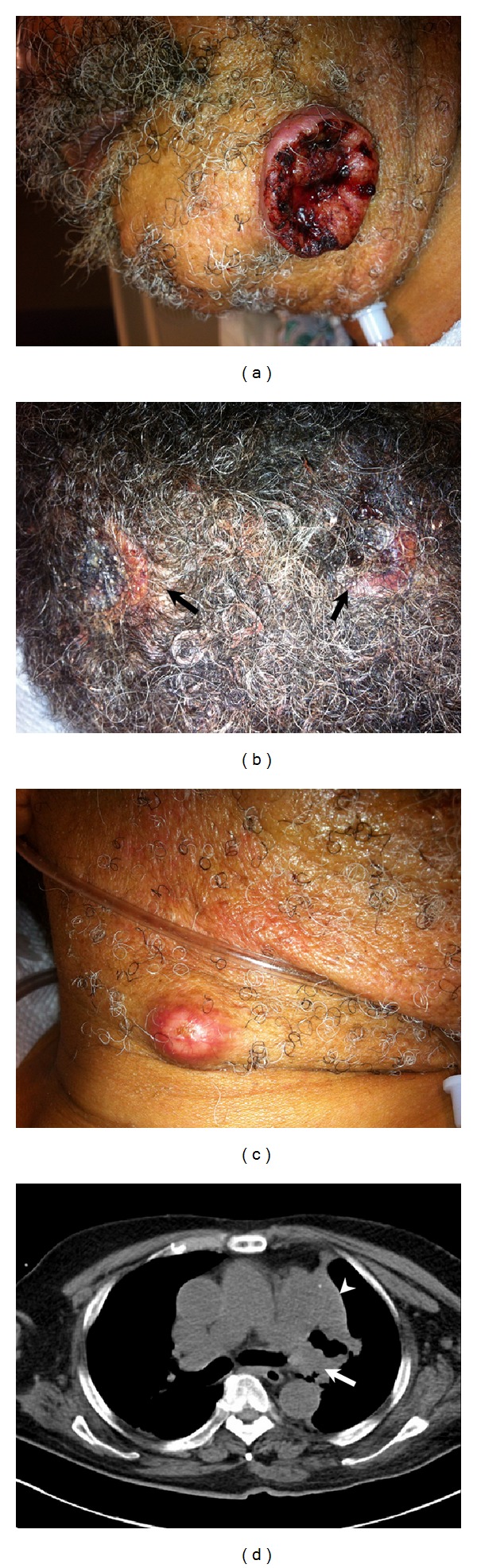
Skin finding revealed multiple large, firm, ulcerative skin lesions with hyperemic centers and elevated rolled edge over the left side of the chin (a) and the scalp (b). A nodular lesion also was seen on the right side of the neck (c). Computed tomography of the chest shows a 5 cm large centrally located left upper lobe mass (arrow) resulting in constrict of the left upper lobe bronchus and left upper lobe atelectasis (arrow head), associated with left hilar adenopathy.

A 70-year-old male patient was admitted to the hospital with two-month history of progressive worsening cough and sputum production associated with exertional shortness of breath. His condition was combined with decline in his functional activity, malaise, loss of appetite, and loss of weight of about 30 Ibs. He also noticed painless ulcerating skin lesions, progressively increasing in size over his face, neck, and the scalp. His past medical history was significant for hypertension and Alzheimer's dementia. He had 60-pack-year history of smoking. On examination, vital signs were within normal limits. BMI was 17.1. He appeared lethargic with temporal and facial muscle wasting. Skin finding revealed multiple large, firm, mobile, nontender, ulcerative skin lesions with hyperemic centers and elevated rolled edges, which were located on the left side of the chin and scalp areas (Figures 1(a) and 1(b)). He also had a nodular lesion on the right lower face (Figure 1(c)) and a black hairy tongue. Chest examination showed diminished air entry with dullness to left upper chest. Laboratory tests showed WBC 11.5 × 10^9^/L and hemoglobin 11.5 g/dL. The chest radiograph (CXR) showed abnormal soft tissue fullness at the left hilum. Computed tomography of the chest (CT) revealed a 5 cm large centrally located left upper lobe mass resulting in constriction to the left upper lobe bronchus and left upper lobe atelectasis (Figure 1(d)), associated with left hilar adenopathy. Bronchoscopy showed that a mass was visualized at the junction of the left upper lobe, and the lingula, completely occluding the entry to the left upper lobe and biopsies were obtained. Skin biopsy was performed, and the result was consistent with poorly differentiated squamous cell carcinoma and was identical to the histopathology of the left lung mass. Overall, the patient's prognosis appeared very poor. The patient refused any further workup or intervention and passed away a few days after discharge.

## 3. Discussion

Lung cancer is a common neoplasm affecting men and women usually after the age of fifty. Only early stage operable disease can be cured, while the remainder of patients develop distant metastases and die. Most common sites of metastasis include the bones, liver, adrenal gland, and brain, while the skin is rarely affected. The percentage of patients with lung cancer that develop skin metastases ranges from 1 to 10%. This must be ruled out in patients with suspicious skin lesions and history of smoking or lung cancer. The diagnosis is usually made using clinical information, and skin biopsy should be obtained for histological examination. All histological types of lung cancer may metastasize to the skin [1]. The incidence of cutaneous metastasis is highest among patient with large-cell carcinoma (2.5%) and low for squamous (0.7%) and small-cell carcinoma (0.3%) [1]. In about 20–60% of cases, the skin lesions are present before or at the same time with the diagnosis of the primary lung tumor [2]. Any body skin area can be involved with metastasis but lung cancers commonly involve the anterior chest, abdomen, and head/neck [3]. Skin lesions have been described in the literature as nodular or ulcerated, mobile or fixed, hard or flexible, single or multiple, and painless lesions [2]. Treatment of solitary cutaneous metastases usually includes surgery alone or combined with chemotherapy, and/or radiation [2]. In the majority of the cases, skin metastases usually present with other internal metastases. If multiple skin lesions or internal metastases exist, chemotherapy is the primary option. Overall, skin metastases and their primaries in the lung are usually incurable and suggest a very poor prognosis. Mean survival is usually about 5-6 months [2, 4]. 

## 4. Conclusion

Skin metastasis from lung cancer is an unusual event carrying an ominous prognosis. A particular high index of suspicion of skin metastases is required in individuals who present with suspicious skin lesions and history of smoking or lung cancer. All types of lung cancer may spread to the skin, and clinical lesions are variable.
